# Clearance of cerebral Aβ in Alzheimer’s disease: reassessing the role of microglia and monocytes

**DOI:** 10.1007/s00018-017-2463-7

**Published:** 2017-02-14

**Authors:** Leah Zuroff, David Daley, Keith L. Black, Maya Koronyo-Hamaoui

**Affiliations:** 1 Department of Neurosurgery, Maxine Dunitz Neurosurgical Institute, Cedars-Sinai Medical Center, 127 S. San Vicente, AHSP A8115, Los Angeles, CA 90048 USA; 2 Perelman School of Medicine at the University of Pennsylvania, Philadelphia, PA 19104 USA; 3 Department of Biomedical Sciences, Cedars-Sinai Medical Center, Los Angeles, CA 90048 USA

**Keywords:** Neurodegenerative diseases, Amyloid-β protein, Aβ-degrading enzymes, Innate immune cells, Myelomonocytes, Phagocytosis

## Abstract

Deficiency in cerebral amyloid β-protein (Aβ) clearance is implicated in the pathogenesis of the common late-onset forms of Alzheimer’s disease (AD). Accumulation of misfolded Aβ in the brain is believed to be a net result of imbalance between its production and removal. This in turn may trigger neuroinflammation, progressive synaptic loss, and ultimately cognitive decline. Clearance of cerebral Aβ is a complex process mediated by various systems and cell types, including vascular transport across the blood–brain barrier, glymphatic drainage, and engulfment and degradation by resident microglia and infiltrating innate immune cells. Recent studies have highlighted a new, unexpected role for peripheral monocytes and macrophages in restricting cerebral Aβ fibrils, and possibly soluble oligomers. In AD transgenic (ADtg) mice, monocyte ablation or inhibition of their migration into the brain exacerbated Aβ pathology, while blood enrichment with monocytes and their increased recruitment to plaque lesion sites greatly diminished Aβ burden. Profound neuroprotective effects in ADtg mice were further achieved through increased cerebral recruitment of myelomonocytes overexpressing Aβ-degrading enzymes. This review summarizes the literature on cellular and molecular mechanisms of cerebral Aβ clearance with an emphasis on the role of peripheral monocytes and macrophages in Aβ removal.

## Introduction

Alzheimer’s disease (AD) is a severe neurodegenerative disorder and the most common form of senile dementia, affecting over 5 million in the United States and 45 million worldwide [1, 2]. AD manifests as a progressive decline in cognitive function and behavior, invariably leading to death [3]. The epidemic of AD is especially damaging to the growing elderly population and the economy that supports them. This immense psychosocial and public health burden calls for a clearer understanding of disease pathophysiology to facilitate the development and implementation of more effective treatment strategies.

Over the past century, our understanding of the molecular mechanisms underlying the development of AD has greatly expanded. Though still pathological hallmarks, extracellular plaques and intracellular neurofibrillary tangles (NFTs) within the brain [4], comprised, respectively, of amyloid-β protein (Aβ) and hyperphosphorylated tau (pTau), no longer describe all pathogenic forms of these proteins. Beyond intracellular threads and tangles, misfolded tau may form extracellular assemblies that propagate through and disrupt synaptically dense regions [5, 6]. Meanwhile, extracellular and intracellular oligomers of Aβ were also found to be highly synaptotoxic and exist in a highly dynamic equilibrium between the small, soluble forms and the larger, insoluble intermediates and fibrils [7, 8]. Recent exploration of this disease outside the brain, in another central nervous system tissue, has further revealed Aβ pathology in the retina of AD patients, including those at early stages [9–13]. Converging data from genetic, physiologic, biochemical, and clinical studies demonstrate a strong association between Aβ accumulation and neuroinflammation, synaptic loss, impaired neuronal function, and ultimately, debilitating cognitive decline [3, 14]. Progressive accumulation and aggregation of Aβ peptides in the brain are thought to be a net result of imbalance between their production and clearance [15]. Moreover, the dramatic increase in cerebral Aβ far precedes the clinical impairment, beginning as early as 20 years prior to symptom manifestation [16]. Therefore, a common view is that any strategy that reduces Aβ levels in the brain, either by inhibiting its production/aggregation or by increasing its clearance, will be advantageous in preventing the development of AD.

Aβ denotes a group of endogenous peptides, typically of 36–43 amino acids. It derives from a larger transmembrane protein, the amyloid precursor protein (APP), in a complex proteolytic process, described extensively elsewhere [17]. The disease-associated (amyloidogenic) aggregation-prone Aβ1-40 (Aβ_40_) and Aβ1-42 (Aβ_42_) alloforms are generated through a sequential cleavage of APP by a β-secretase (BACE1) and a γ-secretase transmembrane complex. Mutations within the gene encoding APP and its Aβ coding sequence were found to cause early-onset, autosomal-dominant inherited forms of familial AD (FAD) [18]. Similarly, patients with Down syndrome (trisomy 21) who carry three copies of the *APP* gene develop AD-like Aβ and tau neuropathology, leading to cognitive decline [19]. In addition, inheritance of mutations within the genes encoding for presenelin-1 and -2 (PS1 and PS2), two components of the γ-secretase complex, invariably lead to FAD [20–22] (Table 1). These rare mutations and haplotypes result in either overproduction or increased aggregation of Aβ, and importantly, in favored generation of the more pathogenic Aβ_42_ alloforms [4, 23]. These findings strongly tie Aβ to the etiology of AD. Further support for this notion came recently from the identification of a protective *APP* mutation in non-demented Icelanders [24]. The A673T mutation in *APP* (alternatively called A2T mutation in Aβ) was shown to reduce amyloidogenic Aβ production and aggregation, providing protection against age-associated cognitive decline [24, 25].

**Table 1:** Genes associated with Alzheimer’s disease

Gene	Type	FREQ^a^	Risk	Locus	Variants	↑ Aβ prod.	↓ Aβ clear.	Effects on Aβ^b^	References
*APP*	FAD^c^	Rare		21q21.3	Mutations Trisomy 21	✓	–	↑ Aβ_42/40_ ratio; ↑ Aβ_42_ aggregation	[4, 18, 21, 26–28]
*PSEN1*	FAD^c^	Rare		14q24.3	Mutations	✓	–	↑ Aβ_42/40_ ratio	[20, 21, 23, 26, 28–31]
*PSEN2*	FAD^c^	Rare		1q42.13	Mutations	✓	–	↑ Aβ_42/40_ ratio	[21–23, 26, 28, 31]
*ABCA7*	LOAD	16%		19p13.3	rs3764650 rs3752246 rs4147929	✓	✓	Understudied; ↑ Aβ secretion; ↓ MΦ/MG Aβ phagocytosis	[21, 32–39]
*ADAM10*	LOAD	Rare		15q21.3	Q170H R181G	✓	–	↑ Aβ production; ↓ α-secretase activity	[40–43]
*ACE*	LOAD	33–48%		17q23.3	Indel; rs4219 rs1800764 rs4343	–	✓	Controversial; ↓ Aβ degradation; ↑ Aβ levels	[43–50]
*APOE4* ^d^	LOAD	3%^e^		19q13.2	ε4 Allele^f^ ε2 Allele^g^	–	✓	↓ Chaperone-mediated Aβ processing, clearance	[51–56]
*BIN1*	LOAD	45%		2q14	rs744373 rs7561528	✓	✓	↑ Aβ production; May ↓ MΦ Aβ phagocytosis	[33, 34, 57–61]
*CD2AP*	LOAD	3%		6p12	rs9296559 rs9349407	–	✓	↑Aβ plaque burden; ↓ Endosome/lysosome clearance	[33, 37, 57, 62, 63]
*CD33*	LOAD	30%		19q13.3	rs3865444^g^ rs3826656	–	✓	↓ Mo/MG Aβ phagocytosis	[33, 34, 64–67]
CLU	LOAD	38%		8p21-p12	rs9331896	–	✓	↓ Chaperone-mediated Aβ clearance	[32, 68–75]
*CR1*	LOAD	20%		1q32	rs3818361 rs6656401 rs6701713	–	✓	↓ Immune-mediated Aβ clearance; ↑ Aβ_42_ levels	[33, 34, 76–79]
*EPHA1*	LOAD	34%		7q34	rs11771145^g^ rs11767557^g^	–	✓	Understudied; ↓ Immune-mediated Aβ clearance	[32–34, 80–82]
*PICALM*	LOAD	36%		11q14	rs3851179^g^ rs541458^g^	✓	✓	↓ Trafficking of Aβ across BBB; ↑ Aβ production	[83–89]
*SIRT1*	LOAD	–		10q21.3	–	✓	✓	↑ MG-dependent Aβ toxicity; ↓ α-secretase activity	[32, 90–95]
*SORL1*	LOAD	4%		11q23.2-q24.2	rs12285364 rs2070045 rs2282649	✓	✓	↑ Aβ production; ↓ APP trafficking to endosomes	[94, 96–101]
*TREM2*	LOAD	6%		6p21.1	rs75932628	–	✓	Controversial; ↓ Mo phagocytosis and immune response	[102–108]

*ABCA7* ATP-binding cassette, sub-family A (ABC1), member 7, *ACE* angiotensin-converting enzyme, *APOE* apolipoprotein E, *APP* amyloid precursor protein, *BIN1* bridging integrator 1, *CD2AP* CD2-associated protein, *CD33* sialic acid-binding immunoglobulin-like lectin 3, *Clear*. clearance, *CLU* clusterin (apolipoprotein J), *CR1* complement component (3b/4b) receptor 1, *EPHA1* EPH receptor A1, *Exp*. gene expression levels in AD, *FAD* early onset familial AD: inherited in an autosomal dominant fashion, *Load* late onset AD, *Mo*/*MΦ* monocytes/macrophages, *MG* microglia, *PICALM* phosphatidylinositol binding clathrin assembly protein, *Prod*. production, *PSEN1* presenilin 1, *PSEN2* presenilin 2, *SIRT1* sirtuin 1, *SNPs* single nucleotide polymorphisms, *SORL1* sortilin-related receptor 1, *TREM2* triggering receptor expressed on myeloid cells 2

^a^Approximate frequency

^b^Postulated effects on Aβ and related immune response

^c^Rare variants identified in LOAD

^d^Strongest genetic risk factor for LOAD

^e^Carriers of one or two *APO*ε*4* alleles

^f^Dose-dependent effect of Apoε4 alleles

^g^Reduced risk for AD

While FAD represents approximately 5% of all AD cases, the remaining majority of AD cases manifest later in life (typically over 65 years of age), and are termed sporadic or late-onset AD (LOAD). The etiology of LOAD is multifactorial: multiple genetic and environmental factors likely contribute to the development of disease. Strong support for the role of Aβ accumulation in both AD forms came from several clinical studies. While in FAD cases cerebral Aβ increase was explained by Aβ_42_ overproduction [109], deficient Aβ_42_ clearance was shown in the brains of LOAD patients [110]. Despite differences in etiology, FAD and LOAD are neuropathologically indistinguishable and present with similar clinical phenotypes [4].

Growing evidence indicates that Aβ exerts its neurotoxic effects in both an alloform- and conformation-dependent manner [7]. Small, soluble oligomeric forms of Aβ_42_ were shown to be especially neurotoxic [111–113] and more strongly predict cognitive decline than Aβ plaque load [114, 115]. Specifically, Aβ oligomers were shown to impact long-term potentiation, synaptic signaling and plasticity, dendritic morphology, and cognition in rodent models [113, 116–119]. Additionally, Aβ was shown to impair neuronal glucose transport [120] and accumulate within mitochondria [121], disrupting vital enzymatic activity and increasing free radical production [122]. Aβ fibrils can also induce inflammatory processes by binding to and activating microglia [123, 124] and peripheral monocytes [125–127]. This toxic microenvironment was further associated with impaired calcium regulation and energy metabolism throughout CNS tissues [128]. Beyond amyloid pathology in brain parenchyma, AD patients frequently exhibit cerebral amyloid angiopathy (CAA) along with reduced cerebral blood flow that can further compromise cognitive capacity [129]. This phenomenon was also found in retina microvasculature [13, 130]. In murine models of AD, it was recently found that vascular amyloid deposits hardened blood vessel walls and reduced blood flow [131].

Although the existence of Aβ plaques and NFTs establishes the definitive diagnosis of AD, many researches have challenged the predominant belief that Aβ is central to the development of disease. For example, studies have demonstrated that NFT pathology correlates more strongly than amyloid plaque load with brain atrophy and cognitive decline [132, 133]. In addition, clinical trials targeting cerebral Aβ plaque removal in symptomatic patients have largely failed to provide a clinical benefit and have consequently raised concerns regarding the role of Aβ in the etiology and treatment of AD [134]. Alternative theories of AD pathogenesis have also been postulated. For instance, different groups consider AD to be a combination of multiple disorders of diverse etiology [135], a by-product of normal aging [136, 137], or initiated by faulty immune activation [138]. Others have described AD as a metabolic disorder similar to diabetes, and even coined the term Type 3 Diabetes to highlight their shared molecular and cellular disturbances, such as insulin resistance, oxidative stress, and glycogen synthase kinase 3β activation [139]. These data are essential as the field continues to both expand and refine our understanding of AD pathogenesis and explore potential therapeutic avenues. However, this evidence does not preclude Aβ from playing a principal role in disease. Indeed, several studies have demonstrated that the presence of misfolded Aβ is sufficient to induce pTau and NFTs *in vitro* and *in vivo* [140–143]. Furthermore, overwhelming data from preclinical animal models have shown that targeting the production, aggregation, or immune-based removal of Aβ, and especially soluble Aβ_42_, preserved synapses and neuronal function as well as prevented cognitive decline [10, 144–146]. Importantly, a recent promising phase Ib human clinical trial, using a monoclonal antibody (aducanumab) to target the removal of both soluble oligomeric and fibrillar Aβ, has reinvigorated the field of Aβ-centered AD therapeutics. After 1 year of monthly aducanumab infusions, patients with prodromal or mild AD displayed a reduced cerebral Aβ plaque load and, by preliminary analyses, exhibited slowing of cognitive decline [147]. Taken together, it is no surprise that Aβ, in its various forms, remains the focus of AD research and a target for AD prevention and therapy.

**Fig. 1:**
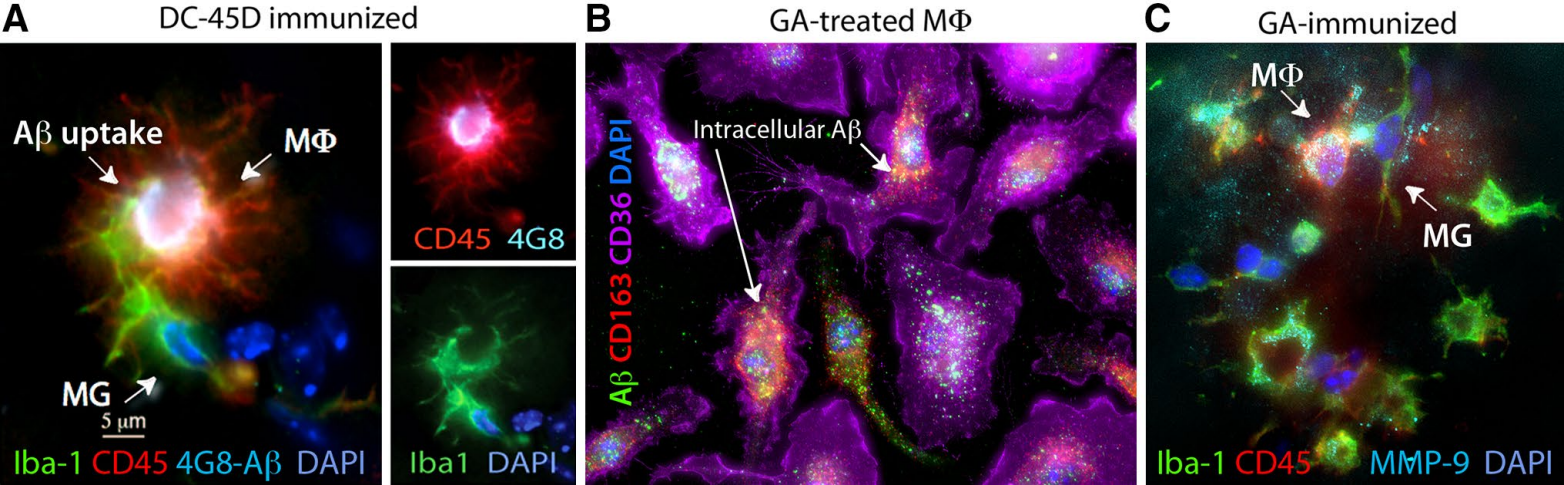
Cerebral Aβ clearance by peripheral monocyte-derived macrophages. **a** ADtg mice were immunized with dendritic cells (DCs) pulsed with an altered myelin-derived peptide (MOG45D). Brain-resident microglia (MG, Iba1^+^/CD45^int-low^), and moreover, blood-borne infiltrating Iba1^+^/CD45^high^ macrophages (MΦ, *red*), are involved in the uptake of cerebral Aβ (4G8^+^; *bright white areas*), as shown in the hippocampal region from an immunized ADtg mouse. Image adopted from Koronyo-Hamaoui et al., J Neurochemistry [148]. **b** Phagocytosis of fibrillar Aβ_42_ (6E10) and co-localization within CD163^+^CD36^high^ bone marrow-derived macrophages in cultures treated with glatiramer acetate (GA). **c** A GA-immunized ADtg mouse brain exhibiting increased expression of Aβ-degrading enzyme (MMP-9) by recruited blood-borne MΦ surrounding Aβ plaques. Microscopic images from Koronyo et al., Brain, [144]

In this review, we summarize various cellular and molecular, physiologic mechanisms of Aβ removal from the brain. Specifically, we cover Aβ transport across the blood–brain barrier (BBB), glymphatic clearance, cellular uptake, and enzymatic degradation. Large-scale genetic studies have further cemented the connection between Aβ accumulation, clearance by innate immune cells, and disease risk, and will be the topic of the following section. Finally, we place a particular emphasis on the growing evidence supporting a key role for microglia, and moreover, monocyte-derived macrophages in the physiological clearance of cerebral Aβ (see Fig. 1), and we examine their potential as targets for disease-modifying therapies.

## Genes related to Aβ homeostasis and Alzheimer’s disease

Historically, the study of AD-related genes pertained to the rare, inheritable and early onset forms of the disease (termed FAD) [4, 22]. These early genetic studies identified FAD as a monogenic disorder resulting from mutations in *APP, PS1*, or *PS2* leading to the amyloidogenic processing of APP and overproduction of synaptotoxic Aβ_42_ (Table 1) [4, 23]. In contrast, the far more common, late-onset AD (LOAD) is a multifactorial disease, with complex and heterogeneous interactions between genetic and environmental factors underlying its development [149, 150]. Importantly, insufficient cerebral Aβ clearance is thought to drive LOAD pathogenesis [110]. The strongest known susceptibility locus for LOAD encodes apolipoprotein E (ApoE) [51, 151]. Carriers of a single, and moreover, carriers of double *APOE4* alleles have a significantly increased risk of developing AD [51, 151]. Apoε4 has been implicated in Aβ trafficking and neurovascular function, with possible additional effects on myeloid cell phenotype and ability to phagocytose Aβ (discussed further below) [52, 152–154]. Recent genome-wide association studies (GWAS), case-control and family-based studies, whole exome sequencing studies, and meta-analyses of large LOAD patient datasets have further identified over 20 novel risk factors with varying effect sizes and frequencies in the population (Table 1) [32–34, 76, 83, 96, 155]. Remarkably, a vast majority of these risk genes are associated with Aβ processing or trafficking as well as with a wide range of immunological responses, especially those related to myeloid cell-mediated Aβ clearance [156]. More specifically, LOAD risk genes have been demonstrated to impact inflammation (*APOE, INDPP5D, CR1, TREM2, MS4A*), complement activation (*CLU, CR1*), the HLA gene complex (*HLA-DRB1, HLA-DRB5*), and myeloid cell-mediated Aβ proteolysis (*ACE, CD2AP*) and phagocytosis (*APOE, BIN1, INPP5D, CR1, ABCA7, TREM2*) [21, 156]. In particular, polymorphisms in the genes *CD33* (sialic acid-binding immunoglobulin-like lectin 3) and *TREM2* (triggering receptor on myeloid cells 2) directly link impaired microglial and macrophage phagocytosis of Aβ to increased susceptibility to AD. The reported effect size of *TREM2* variants on AD risk has varied in the literature [96, 155]: some investigations estimate an odds ratio of 3–4 (similar to the risk of carrying a single Apoε4 allele), while others show only a small to moderate effect [96, 151, 155, 157]. Nonetheless, TREM2 has remained in the spotlight for its effects on myelomonocytic cell phenotype and Aβ phagocytosis, which will be discussed in later sections. It has long been questioned whether AD-associated inflammation and myeloid cell dysfunction drive disease pathogenesis or instead represent a subsequent reaction to the associated neuropathology [123, 158]. Yet, these recent large-scale genetic studies, compiling data from thousands of AD subjects, illustrate unequivocally the principal role of immunological processes in development of AD, and for the first time, provide genetic evidence supporting the significance of the peripheral immune system.

## Mechanisms of Aβ clearance

The key mechanisms of Aβ clearance were shown to involve either Aβ removal to the peripheral blood and lymphatic systems or degradation within the CNS tissues. Aβ reaches the peripheral circulation via chaperone-mediated transport across the blood brain barrier (BBB) [159], perivascular drainage [160], or through the glymphatic system [161, 162]. In the parenchyma, myelomonocytic cells were shown to phagocytose fibrillar Aβ, and perhaps their soluble oligomeric forms as well. These professional phagocytes, together with astrocytes and neurons, are jointly responsible for degradation and removal of amyloidogenic Aβ alloforms [123, 163]. Though each system likely contributes to Aβ clearance to varying extents, their summed effects are essential for Aβ homeostasis. This implies that perturbations of any singular process may underlie or predispose to pathologic Aβ accumulation, and consequently development of AD.

## Extracellular enzymatic degradation of Aβ

Secreted peptidases are critical for the catabolism of Aβ peptides. These enzymes were reported to have an affinity for specific domains within the Aβ amino acid sequence and an ability to cleave and convert these peptides to shorter, more benign forms [164–167]. Table 2 describes major Aβ-degrading enzymes, their substrates, their cellular location and the cell types known to express and secrete them. The following paragraphs describe several Aβ-degrading enzymes that have been central in AD research.

**Table 2:** Amyloid β-degrading enzymes in Alzheimer’s disease

Enzyme	Type	Expression	Active site	Aβ substrate	References
NEP	Type II integral membrane zinc metalloprotease	Membrane-bound; neurons, Mo/MΦ, MG, astrocytes	Ext	sAβ_40,42_	[166, 168–173]
IDE	Zinc metalloprotease	Cytosolic, cell surface, secreted; neurons, Mo/MΦ, MG, astrocytes	Ext and Int	sAβ_40,42_	[165, 174–177]
MMP-2	Matrixin; zinc metalloprotease	Membrane-bound, secreted; endothelial cells, Mo/MΦ, pyramidal neurons, astrocytes	Ext	sAβ	[178–182]
MMP-3	Matrixin; Zinc metalloprotease	Secreted; endothelial cells, Mo/MΦ, MG, astrocytes	Ext	sAβ	[183, 184]
MMP-9	Matrixin; zinc metalloprotease	Secreted; neurons, MG, astrocytes, Mo/MΦ	Ext	sAβ; fAβ; Mature plaques	[144, 167, 185–190]
ACE	Zinc metalloprotease	Membrane-bound, Secreted; Muscle and endothelial cells, lymphocytes and Mo/MΦ	Ext	sAβ_40,42_; fAβ_40,42_	[145, 164, 191, 192]
ECE-1	Zinc metalloprotease	Membrane-bound; endothelial cells, neurons, Mo/MΦ, MG, astrocytes	Ext	SynAβ_40_; Aβ in Ctx and Hip	[193–195]
Cathepsin B	Cysteine protease	Within lysosomes; various cell types	Int	Controversial; APP; Aβ_40,42_	[196–201]
Cathepsin D	Aspartic protease	Within lysosomes; various cell types	Int	sAβ_40,42_	[202–204]

*ACE* angiotensin-converting enzyme, *APP* amyloid precursor protein, *Ctx* cortex, *ECE-1* endothelin-converting enzyme 1, *Ext*. extracellular, *fAβ* fibrillar Aβ, *Hip* hippocampus, *IDE* insulin-degrading enzyme, *Int*. intracellular, *Mo*/*MΦ* monocytes/macrophages, *MG* microglia, *MMP-2* matrix metalloproteinase 2, *MMP-3* matrix metalloproteinase 3, *MMP-9* matrix metalloproteinase 9, *NEP* neprilysin, *oAβ* oliogomeric Aβ, *sAβ* soluble Aβ, *SynAβ* synthetic Aβ

### Angiotensin-converting enzyme (ACE)

ACE is a zinc-dependent peptidase with significant expression by endothelium throughout the body as well as by cortical neurons in the brain [205]. Most well known for transforming angiotensin-I to angiotensin-II and for its role in regulating hemodynamic stability and salt balance, ACE was also shown to degrade Aβ, and importantly, cleave Aβ_42_ into the less toxic Aβ_40_ alloform [164]. In post-mortem analyses, cortical and perivascular ACE expression was upregulated in the brains of AD patients and correlated with parenchymal plaque load and extent of perivascular amyloid deposition, respectively [206, 207]. Furthermore, lower levels of ACE protein and its activity were associated with lower CSF Aβ, indicating more prominent amyloid pathology in the parenchyma [44]. It was thus hypothesized that increased ACE activity in CNS tissues is a protective response to increasing amyloid pathology. While this claim is partially supported by both genetic studies in humans and physiologic studies in ADtg mice, there are inconsistencies within the literature. Both case-control studies and several large meta-analyses have identified an insertion within intron 16 of the gene *ACE1* that reduces plasma ACE levels and increases risk for AD [45–47]. However, these findings were not always replicated [208]. Interestingly, AD patients homozygous for the insertion polymorphism had a greater risk of cognitive deterioration and clinical progression than other ACE genotypes [48], suggesting ACE activity may critically modulate the pathophysiology underlying neurodegeneration. Indeed, one long-term study of the ACE-inhibitor (ACE-I) captopril in ADtg mice supports the role of ACE in Aβ clearance, as both Aβ plaque load and Aβ_42_ levels were elevated after 11 months of treatment [209]. It is important to note, however, that studies of shorter duration did not report a measurable effect of other ACE-Is on Aβ pathology [205]. In ADtg mice, ACE overexpression by microglia and monocytes/macrophages lead to a dramatic reduction in cerebral Aβ levels and cognitive decline [145, 191], demonstrating great therapeutic potential discussed further below. Taken together, there is substantial, although inconsistent, evidence implicating ACE in the physiological clearance of Aβ that merits further investigation.

### Insulin-degrading enzyme (IDE)

IDE is a zinc metalloprotease that is capable of degrading soluble Aβ_40_ and Aβ_42_ into non-toxic fragments [165, 174]. Although primarily localized in the cytosol, a small fraction of IDE is secreted by glial cells [175, 176, 185] or expressed on the cell surface of neurons [177], where it serves as a critical enzyme for extracellular Aβ degradation [165, 210]. Investigations in human and AD rodent models have yielded varying evidence regarding IDE mRNA expression, protein levels, and activity in the AD brain, most likely because the behavior of IDE is highly dependent on age [211–213], brain region [211–213], disease severity [212, 213], and *APOE* status [214]. In general, it seems IDE levels and activities are upregulated in response to Aβ exposure, with the exception of *Apoε4*/*4* carriers, who exhibit reduced IDE expression.

### Matrix metalloproteinase-9 (MMP-9)

MMP-9 is a secreted enzyme and member of the zinc metalloprotease (MMP) family. In general, MMPs are responsible for the degradation and maintenance of the extracellular matrix. MMP-9 has been shown to degrade compact plaques [186, 187] as well as soluble Aβ_42_ and Aβ_40_ [167]. In the CNS, MMP-9 is expressed by neurons [188], microglia [189], astrocytes [190], and infiltrating Iba^+^/CD45^hi^ monocytes (Fig. 1C) [144, 148]. MMP-9 has also been shown to act as an α-secretase, favoring non-amyloidogenic processing of APP and the production of sAPPα [215]. In addition to its efficient degradation of Aβ, MMP-9 was shown to be involved in both TNFα-mediated pro-inflammatory and anti-inflammatory signaling in activated macrophages and microglia [216, 217]. Elevated levels of MMP-9 have been correlated with BBB breakdown, demyelination, and cell death in other CNS disorders like multiple sclerosis [218] and spinal cord injury [219]. These effects should be considered when modulating MMP-9 activity *in vivo*.

### Neprilysin (NEP)

NEP is a type II integral membrane zinc metalloprotein with the bulk of its structure, including the active site, facing the extracellular space. NEP is expressed throughout the brain, predominantly on pre- and post-synaptic neuronal membranes [168, 169], and by microglia [170] and astrocytes [171]. NEP is considered the most potent Aβ-degrading enzyme [220, 221], preferentially cleaving oligomeric Aβ_42_ and Aβ_40_ [166, 172] but not fibrillar forms. NEP expression and activity has been shown to decline with age and disease in post-mortem human AD brain tissue [222], which may contribute to Aβ accumulation. Modeling this reduction by dampening NEP expression [223, 224] or activity [225] in ADtg mice resulted in elevated Aβ pathology and cognitive deficits. Conversely, the beneficial effects of NEP overexpression speak to the therapeutic potential of targeting neprilysin activity, discussed further below [146, 172].

### Enzymatic degradation by innate immune cells

NEP, IDE, ACE and MMP-9 are Aβ-degrading enzymes expressed by innate immune cells and represent a crucial pathway by which these cells may eradicate pathogenic Aβ (Table 2). Expression of NEP, IDE, and MMP-9 was shown to decline in microglia of aged APP/PS1 mice, which may contribute to their functional impairment in later stages of AD [170]. This altered microglial phenotype was contingent on the presence of Aβ, as microglia from age-matched controls did not exhibit reduced enzyme expression. Microglial expression of NEP and IDE were also shown to be highly inducible *in vitro* and correlated with enhanced clearance of soluble Aβ_42_ [226].

For proteolytic processing of Aβ by monocyte-derived macrophages, the expression of MMP-9 appears to be especially important. APP_SWE_/PS1_ΔE9_ mice infused with CD115^+^ monocytes or immunized with the altered myelin-derived antigens, such as glatiramer acetate (GA) or myelin oligodendrocyte glycoprotein-derived peptide (MOG45D) displayed increased accumulation of MMP-9-secreting macrophages surrounding Aβ plaques (Fig. 1c), along with a marked reduction in Aβ neuropathology and cognitive impairment [144, 148]. GA stimulation of bone marrow-derived macrophages *in vitro* also dramatically induced MMP-9 expression [144]. Additionally, peripheral macrophages cultured on top of plaque-bearing brain sections of PDAPP mice cleared Aβ, in part, by upregulated expression of MMP-9 [185]. Interestingly, macrophages expressed MMP-9 in an ApoE-dependent manner. Apoε4 significantly dampened MMP-9 expression, suggesting an additional mechanism by which Apoε4 disrupts Aβ clearance [185].

Furthermore, ACE has a demonstrated ability to modulate the behavior of innate immune cells in ADtg murine models [191, 227]. In other disease models, targeted overexpression of ACE to myelomonocytic cells enhances their immune function, including their ability to clear cellular debris and promote tissue repair. Targeted ACE overexpression to myelomonocytes (ACE10/10 model) introduced to APP_SWE_/PS1_ΔE9_ transgenic mice resulted in increased infiltration of monocyte-derived macrophages that were tightly associated with Aβ plaques and displayed increased ability to phagocytose Aβ [145]. The net result was reduced soluble and insoluble Aβ levels, attenuated neuroinflammation, and improved cognitive performance. In contrast, inhibition of ACE catalytic domains in ACE10/10-ADtg mice exacerbated cerebral Aβ pathology [145]. Overall, the beneficial outcomes of ACE overexpression in myelomonocytes were most likely due to the summed effects of the enhanced immune response and proteolytic capacity endowed by ACE expression.

## Intracellular degradation systems

Another important mechanism of Aβ catabolism is undertaken within cells that either absorb or engulf Aβ forms. Three such critical pathways—autophagy, endosomal/lysosomal degradation, and the ubiquitin–proteasome system (UPS)—prevent intracellular protein aggregation, and are thus instrumental in protecting against the neurotoxicity of cytosolic Aβ accumulation. In AD brains, however, these systems are considerably compromised [228–231]. Degradation targets for both the UPS and autophagy originate from the cytosol, although their identities differ between the two processes. Autophagy typically facilitates clearance of larger protein aggregates and damaged organelles, while the UPS degrades misfolded or damaged proteins. Furthermore, the UPS is more highly regulated than autophagy, requiring poly-ubiquitination of the target protein for its degradation. The lysosome, too, facilitates intracellular protein degradation, though the origin of these proteins may be either cytosolic or extracellular. Because the lysosome is a final common pathway for several systems, including autophagy, it is discussed separately below.

### Lysosomal degradation

The lysosome is the final destination for both autophagic vacuoles and the endosomes formed by receptor-mediated endocytosis. The latter process occurs in neurons and glia through a distinct set of molecular chaperones, discussed in greater detail in the following sections. Each lysosome contains a cocktail of hydrolytic enzymes capable of degrading Aβ; however, the hydrolytic machinery is often overwhelmed in AD [228, 232–234]. As Aβ load exceeds the degradation capacity of the lysosome, aggregates may grow larger or leak into the cytosol [228, 232–234]. Aging [235] and the presence of Apoε4 [234, 236] particularly promote lysosomal instability. Intracellular Aβ negatively impacts multiple cellular and organelle functions, including proteasome inhibition, mitochondrial abnormalities, tau hyperphosphorylation, and presumably, the seeding of amyloid plaques following cell death [121, 237, 238].

Myeloid cells in particular may suffer AD-associated deficits in endosomal-lysosomal trafficking and Aβ processing. Microglia isolated from plaque-bearing sections of human AD tissue indicated that Aβ fibrils were located in the endoplasmic reticulum and deep invaginations of the cell membrane, instead of within endosomes or lysosomes [239]. Even non-diseased microglia cultured with fibrillar Aβ_42_ showed incomplete intracellular degradation, with non-degraded fibrils remaining in phagosomes for up to 20 days [239, 240]. This impairment was not seen in peripheral macrophage cultures under the same conditions. In fact, after 3 days of incubation with fibrillar Aβ, less than 30% of Aβ was retained in peritoneal macrophages, indicating successful degradation, while 80% remained associated with microglia [241]. One possible explanation for deficient microglial clearance is insufficient activity of lysosomal Aβ-degrading enzymes. In support of this notion, incubating microglia with mannose-6-phosphate tagged lysosomal enzymes rescued the clearance impairment. Mannose-6-phosphate typically targets hydrolytic enzymes to the lysosome from the Golgi apparatus, and this modification has been used to deliver extracellular enzymes to the lysosome in experimental conditions [242].

While healthy peripheral macrophages appear better equipped to degrade fibrillar Aβ than resident microglia, monocytes in AD patients exhibit lysosomal dysfunction [125, 229]. Specifically, more undigested Aβ molecules exist within monocytes isolated from AD patients compared to those from healthy age-matched controls, a deficit partially attributable to reduced expression and activity of cathepsin D and other major lysosomal enzymes [229, 243]. Upregulation of miR-128 was shown to target the transcripts of these enzymes and mediate their suppression. The discrepancy in lysosomal degradation capacity between microglia and infiltrating macrophages highlights their non-redundant roles in restricting Aβ pathology and as targets for future intervention.

### Autophagy-mediated degradation

Three types of autophagy exist: macroautophagy, microautophagy, and chaperone-mediated autophagy (CMA). While both macroautophagy and CMA dysfunction are implicated in AD [244, 245], the former is considered to be the predominant process, and will be referred to simply as “autophagy” throughout [245]. The mechanism of autophagy-mediated clearance involves isolation of cytoplasmic contents by a double-membrane vesicle called an autophagosome or autophagic vacuole (AV). Subsequent lysosomal fusion facilitates degradation of the AV and its contents [246], which may include Aβ and APP [247, 248]. In both AD patients and ADtg models, autophagy is markedly impaired, evidenced by the large accumulation of unprocessed, Aβ-rich AVs in dystrophic neurites [249, 250]. Indeed, deficits in the autophagy-lysosomal pathway occur early in the disease process, perhaps even preceding Aβ accumulation [230, 251]. Reduced expression of key autophagic proteins (beclin-1 and autophagy proteins 5 and 7) likely contribute to autophagic dysfunction, Aβ accumulation, and neuronal cell death [248, 252–254]. Furthermore, perturbations in signaling through the mammalian target of rapamycin (mTOR) pathway, the key regulator of autophagic activity, may also contribute to its impairment in AD. Under nutrient-rich conditions, heightened mTOR signaling suppresses autophagy by phosphorylating proteins necessary for AV formation and elongation [255]. Other pathologic conditions, such as cellular starvation, oxidative stress, organelle damage, and protein aggregation, inactivate mTOR and promote autophagy as a protective response [256, 257]. In the brains of AD patients, however, mTOR signaling was shown to be inappropriately active given the toxic environment [258]. Inhibition of the mTOR pathway has thus emerged as an attractive target for therapeutic intervention, with a demonstrated benefit on Aβ levels and cognition in murine models of AD [259].

### Ubiquitin–proteasome system (UPS)

The UPS is a highly regulated degradation process for cytosolic short-lived and misfolded proteins. As such, it is an important protective mechanism against neurotoxic protein aggregates. Briefly, specific proteins are polyubiquinated by a series of ligases (E1, E2, and E3) for recognition and degradation by the 26S proteasome complex. Whether UPS dysfunction is a cause or consequence of AD-related degeneration remains unknown. In favor of the former, both ubiquitin conjugation and proteasome activity decline with age and in AD tissue [231, 260, 261]. Areas with reduced proteasome function overlap with those greatly impacted by AD: the hippocampus, nearby limbic structures, and the inferior parietal lobe [231]. Diminished activity of the 26S complex promotes Aβ deposition and perhaps its production as well through increased maturation and trafficking of APP [262, 263]. Taken together, this data could imply that declining proteasome function in aging and disease leaves the brain susceptible to Aβ aggregation. Nonetheless, multiple reports have demonstrated that Aβ accumulation, in fact, inhibits proteasome activity, possibly by directly binding to the 20S catalytic subunit [238, 263]. Aβ accumulation may then contribute to proteasome dysfunction rather than result from it, although these interactions need not be mutually exclusive.

## Aβ clearance mediated by extracellular chaperones

Removal of Aβ into the peripheral circulation is thought to facilitate the majority of physiologic Aβ clearance [264]. Transport across the BBB requires a specialized transport system of molecular chaperones. Specifically, members of the LDL receptor (LDLR) family, such as the low-density lipoprotein-related protein 1 (LRP-1) and ATP-binding cassette (ABC) transporters, are primary receptors for Aβ efflux [264]. LRP-1-mediated transport requires the assistance of additional adaptor proteins, and this system in total will be the focus of this section. Transporters that mediate Aβ influx into the brain parenchyma, such as the receptor for advanced glycation endproducts (RAGE), will not be discussed.

### Lipoprotein-related protein 1 (LRP-1)

Located on the abluminal surface of brain endothelial cells, LRP-1 binds either ApoE-Aβ complexes or Aβ alone [53, 265], subsequently stimulating endocytosis of either species. Notably, once Aβ is contained within endothelial cells, the luminal transport protein ABCB1 facilitates the removal of Aβ species into the vascular lumen [266]. Blocking LRP-1 expression in healthy, non-ADtg mice led to impaired Aβ clearance across the BBB, and consequently, greater Aβ deposition and cognitive deficits [267]. This study may recapitulate some of the consequences of declining LRP-1 expression reported in ADtg mice, AD patients, and aging adults [159, 265, 268]. Additionally, LRP-1 is expressed on neurons, astrocytes, and microglia, facilitating cellular Aβ uptake and lysosomal degradation within these cells [269–271].

### Phosphatidylinositol binding clathrin assembly protein (PICALM)

PICALM is expressed on endothelial cells, and to a lesser extent, on neurons [272]. PICALM primarily functions as an adapter protein for the transcytosis of the Aβ-LRP-1 complex across the BBB. In addition to its role in Aβ clearance, recent reports show that single nucleotide polymorphisms (SNPs) in the upstream coding region for PICALM are major risk factors for AD [83, 273]. This may indicate that appropriate PICALM function is protective. In support of this, PICALM levels in cortical microvessels of subjects with advanced AD were half the levels measured in age-matched controls. Subjects with the lowest PICALM levels displayed the greatest Aβ burden and cognitive impairment [274].

### Apolipoprotein E (ApoE)

Under physiologic conditions, ApoE is a carrier protein that maintains cholesterol and phospholipid homeostasis [275]. Major ApoE receptors include LDLR, LRP-1, the very low-density lipoprotein receptor (VLDLR), and ApoE receptor 2 (ApoER2) [276, 277]. However, the exact role of ApoE in AD pathogenesis remains elusive despite mounting evidence from genetic, physiologic, and clinical studies that unequivocally supports the carrier protein’s importance [51, 52, 151, 278–281]. *In vitro* studies have helped to elucidate the role of ApoE, demonstrating that it binds Aβ directly under certain conditions [282]. It is thought that the resulting ApoE-Aβ complexes bind to and are internalized by LRP-1 for delivery to the vasculature and removal from the brain [53, 68]. Supporting this amyloid-clearing role for ApoE, a recent study revealed that ApoE levels inversely correlated with cerebral Aβ load in non-demented healthy controls [283]. In contrast, however, ApoE was shown to compete with fibrillar or soluble Aβ for uptake and degradation by microglia and astrocytes, respectively [54, 284]. Taken together, the literature suggests distinct mechanisms by which ApoE enhances and hinders Aβ clearance. The effect likely depends on the specific Aβ conformation, the ApoE isoform and its lipidation state, as well as the relative ApoE receptor expression on the target cell [278].

Three isoforms of ApoE exist in humans: Apoε2, Apoε3, and Apoε4 [279]. Evidence suggests that the *APOE2* allele may be protective against AD [151]; conversely, carrying one, or to a greater extent, two *APOE4* alleles significantly increases the risk of developing AD and reduces the age of onset [51, 151, 282]. Furthermore, the *APOE4*/*4* genotype is associated with accelerated and more pronounced cerebral amyloid pathology and CSF abnormalities [285]. Several pathogenic mechanisms may explain this increased risk associated with Apoε4. First, the rate of vascular Aβ clearance is diminished in those expressing Apoε4 compared to other isoforms [52, 53, 152], perhaps due to its reduced affinity for Aβ [286]. Additionally, Apoε4 can redirect the ApoE-Aβ complex to a different receptor, VLDLR, which has slower internalization kinetics than other LDLRs [53]. The net result is reduced internalization of Aβ by LRP-1, and ultimately reduced Aβ clearance [280, 281, 287]. Apoε4 may also promote damage to the BBB by upregulation of pro-inflammatory signaling through cyclophilin A [153]. In non-demented murine models, Apoε4 led to reduced cerebral blood flow and microvascular length, while also increasing BBB permeability [153]. A compromised BBB can reduce vascular Aβ clearance and predispose to further injury through leakage of toxic blood proteins [153, 288]. These destructive outcomes were not observed in mice expressing Apoε2 or Apoε3 [153, 289].

Apoε4 may hinder other important mechanisms of Aβ clearance, namely, intracellular catabolism by neurons and innate immune cells. Specifically, Apoε4 may interfere with these processes by inducing lysosomal leakage or by impeding myeloid cell-mediated clearance [185, 234, 236]. Though ApoE normally has an anti-inflammatory effect, this trait is markedly dampened by expression of Apoε4 on innate immune cells [154]. Crossing *APOE4*-targeted replacement mice with the 5XFAD ADtg model greatly increased microgliosis and astrogliosis surrounding Aβ plaques [290]. Similarly, in cell cultures of microglia and astrocytes isolated from these Apoε4-expressing mice, more pro-inflammatory cytokines were released from these cells in response to soluble oligomeric Aβ than from those expressing Apoε3 [154]. Furthermore, peripheral macrophages expressing Apoε4 exhibited a diminished capacity to phagocytose and clear Aβ when cultured on top of plaque-bearing brain sections of PDAPP mice [185]. It remains unclear whether Apoε4 influences AD predominantly through gain of toxic function, loss of protective function (i.e. vascular/immune cell dysfunction), or both. Further investigation is required to reveal the exact role of ApoE and its isoforms in AD, and the possible therapeutic potential of its manipulation.

## Glymphatic clearance

The glymphatic system is a pathway of brain-wide waste clearance for small proteins and metabolites. In this pathway, CSF enters the periarterial space and, driven by arterial pulsations, enters the brain parenchyma to exchange with the interstitial fluid (ISF). Bulk flow of CSF/ISF, containing extracellular molecules such as Aβ, are then driven to perivenous spaces for recirculation in the CSF or clearance to peripheral lymphatics [161, 162]. Glymphatic activity is greatest during sleep, with Aβ clearance rates doubling those observed in periods of wakefulness [291]. The glymphatic system was named, in part, for acting as a surrogate to CNS lymphatic drainage, a system the brain traditionally lacked. However, a recent, seminal study has identified meningeal lymphatic vessels for the first time [292, 293]. This groundbreaking discovery calls for a re-evaluation of current notions of the neuroimmune connection, and raises exciting potential explanations of the pathophysiology of Aβ accumulation and defective clearance in some cases.

Water channels known as aquaporin 4 (AQP4) are the key elements in CSF-ISF exchange, and thus clearance through the glymphatic pathway. AQP4 is located on astrocytic end feet and encircles the vasculature. Mice lacking astrocytic AQP4 showed reduced CSF influx by ~70% and decreased interstitial Aβ clearance by ~55–65% [161, 162]. Advanced age also reduced glymphatic clearance rates in murine models, perhaps due to an age-dependent loss of AQP4 polarization [294]. Interstitial solutes may also be cleared directly into the CSF compartment through periarterial pathways flowing opposite to the glymphatic system. These two pathways may not be mutually exclusive; they might be two components of the same system, or their activities may vary in space and time throughout the CNS [264].

## Myeloid cell-mediated phagocytosis

A growing body of evidence supports the emerging concept that activated inflammatory cells, mainly brain-resident microglia and infiltrating blood-borne monocyte-derived macrophages, are critical for the physiological clearance of Aβ [148, 295–298]. Microglia are tightly associated with Aβ deposits and senile plaques, and early studies have documented their involvement in cellular Aβ uptake [299, 300]. However, these investigations were lacking the capacity to distinguish activated microglia from blood-borne macrophages due to their similar immunophenotype and function. Recruited macrophages were thus inappropriately characterized as part of the microglial pool, and confusion ensued over their unique behavior [296, 301].

Today’s newer methodologies delineate subtle differences in marker expression, allowing for a more accurate categorization and attribution of function to these cell populations. For example, standard CD11b (MAC1), isolectin B4 (IB4), F4/80, or ionized calcium binding adapter molecule 1 (Iba1) markers in the brain are indistinguishably expressed by both infiltrating monocytes and resident microglia [302, 303]. Yet, the combination of one of these myelomonocytic markers with differential expression levels of CD45 [304, 305], P2RY12 [306], or Ly6C [303] can help differentiate these cell types (Fig. 1a, c). Other approaches may involve fluorescent labeling of peripheral innate immune cells (i.e. green or red fluorescent protein-labeled, GFP or RFP, respectively) or introducing genetic modifications, such as targeted NEP- or ACE overexpression in monocytic cells [145, 146, 191].

Other key developmental and functional differences between microglia and macrophages help distinguish these unique cell types. Microglia originate from hematopoietic stem cells of the yolk sac [307], while infiltrating monocyte-derived macrophages originate from bone marrow hematopoietic myelomonocytes [308]. In early post-natal life, microglia participate in synaptic pruning [309]. Later on, they are critical for maintaining CNS homeostasis, regulating immune surveillance, and responding to pathologic changes such as Aβ aggregation [310]. Less is known about CNS monocyte interactions under physiological conditions [311]. A comprehensive comparison of these two cell types is beyond the scope of this manuscript; however, detailed reviews on their unique embryology, development, and immune responses can be found elsewhere [303, 307, 308].

Heterogeneous populations of these immune cells exist in the brain, especially in the diseased state. Their demonstrated clearance capacity varies given the experimental paradigm and the phase of disease studied. Table 3 provides a summary of research on monocytes/macrophages in human AD subjects, while Table 4 briefly describes similar data in rodent models. The discussion that follows describes the phagocytic process mediated by microglia and monocyte-derived macrophages and the conditions in which they differ. When evaluating this data, it is important to keep in mind the difficulties involved in assessing peripheral monocytes and microglia as distinct cell types. Therefore, we cannot rule out the possibility that some investigations illustrating a role for microglia may also include effects of infiltrating monocytes.

**Table 3:** Alzheimer’s disease-related impairments in human myeloid cells

Study type	Study design	Altered protein/gene	Mo phenotype and Aβ clearance	References
HC Mo and MG	Pulse-chase analysis of cytokine impact on Aβ degradation	↑IFN-γ, TNF-α ↑IL-4, IL-10, and TGF-β1	↓ Aβ degradation with pro-inflammatory cytokines; ↓ IDE ↑ Aβ degradation with anti-inflammatory and regulatory cytokines	[312]
AD Mo	rt-PCR and flow cytometry analysis of CD33 expression	↓ CD33 mRNA	↓ CD33^+^ Mo in AD Patients; Positive correlation between number of CD33^+^ Mo and MMSE scores	[313]
	Inflammatory profile; Mo analysis	↑ HLA-DR and CD16 ↑ MCP-1 plasma levels ↓ CCR2 expression	↓ Cerebral recruitment of Mo; ↑ Granularity by SSC	[314]
	Compared Mo from AD patients to HC	↑ Inflammatory profile expressing CCR2, IL-6, IL-23, TLRs ↓ MGAT3 and TLR ↓ Cathepsin B, D, S ↓ Activity of β-Galactosidase, α-Manosidase β-Hexosaminidase	↑ Apoptosis; ↓ Aβ phagocytosis by Mo; Impaired phenotype	[125, 127, 229, 243, 315]
AD vs. MCI Mo	Histone acetylation; cytokine release; susceptibility to cell damage	↑ Production of MIP2 and TNF-α ↓ Acetylation of H4K12 compared to MCI	↑ Mo cell damage susceptibility in AD vs. MCI	[316]
AD peripheral blood	Microarray assessment of gene expression in blood; blood count	Multiple early changes in gene expression >700 altered in blood from MCI, AD vs. HC	↑ Mo number in AD vs. HC; ↑genes encoding cell adhesion molecules and other immune-related genes	[317]

*CCR2* C-C chemokine receptor type 2, *CD33* Sialic acid-binding immunoglobulin-like lectin 3, *H4K12* histone H4 at lysine 12, *HC* healthy control, *HLA-DR* human Leukocyte Antigen–antigen D Related (MHC class II surface receptor), *IDE* insulin degrading enzyme, *IFN-γ* interferon-γ, *IL-4* interleukin-4, *IL-6* interleukin-6, *IL-10* interleukin-10, *IL-23* interleukin-23, *MCI* mild cognitive impairment, *MCP-1* monocyte chemoattractant protein-1, *MG* microglia, *MGAT3* beta-1,4-mannosyl-glycoprotein 4-beta-*N*-acetylglucosaminyltransferase, *MIP2* macrophage inflammatory protein 2, *MMSE* mini-mental state examination (Folstein test)—questionnaire used extensively in clinical and research settings to measure cognitive impairment, *Mo*/*MΦ* monocytes/macrophages, *rt-PCR* reverse transcription polymerase chain reaction, *SSC* side light-scatter characteristics (flow cytometry—measure of granularity and differentiation), *TGF-β1* transforming growth factor-β1, *TLRs* toll-like receptors, *TNF-α* tumor necrosis factor-α

**Table 4:** Studies in rodent models of Alzheimer’s disease implicating a role for peripheral myeloid cells in cerebral Aβ clearance

Study Type	Study design	Mo infiltration^a^	Aβ phagocytosis by Mo	Aβ levels	Neuroinflammation	Cognition	References
BM Transplantation	GFP-labeled BM cells in ADtg	✓	✓	↓	–	–	[144, 146, 295, 318, 319]
Blood Enrichment of BM-derived Mo	Treated ADtg mice with M-CSF or infusion of CD115^+^ GFP-labeled Mo	✓	✓	↓	↓	↑	[144, 146, 320, 321]
Immune Modulation	MOG45D-DC or GA immunization of ADtg	✓	✓	↓	↓	↑	[144, 148, 297]
Genetic Manipulation in Mo/MG	Infusion of GFP-labeled CD11b^+^ WT- or NEP-overexpressing Mo from healthy murine BM donors in ADtg	✓	–	↓	–	–	[146]
	Targeted ACE overexpression of CD115^+^ Mo/MG in ADtg	✓	✓	↓	↓	↑	[145, 191, 227]
	Targeted blockade of TGF-β and Smad2/3 signaling in innate immune cells of ADtg	✓	✓	↓	↓	–	[322]
	Upregulation of TREM2 in ADtg	–	✓	↓	↓	↑	[323]
	*TREM2* knockout in ADtg and stroke models	Χ (CD45^hi^Ly6C^+^)	✓	↓	↓	–	[324, 325]
	SCARA1 upregulation	–	✓	↓	–	–	[326]
	Cultured WT macrophages on plaque-bearing sections of murine models	–	✓ (*APOE* ^b^)	↓	–	–	[185]
	CCL2 (MCP-1) and APP expression effects on Aβ clearance in primary BM-derived macrophages	–	✓	↓	–	–	[327]
Ablation	Depletion of CD11c^+^ BM-derived myeloid cell or perivascular MΦ in ADtg	Χ	Χ	↑	–	–	[296, 297, 328, 329]
Inhibited Mo Infiltration	CCR2-deficient Mo in ADtg	Χ	Χ	↑	–	↓	[298, 330, 331]

*Aβ* amyloid-beta protein, *ACE* angiotensin-converting enzyme, *ADtg* transgenic murine models of Alzheimer’s disease, *APOE* apolipoprotein E, *APP* amyloid-precursor protein, *BM* bone marrow, *CCL2* C-C chemokine ligand 2, alternatively named monocyte chemotactic protein 1 (MCP-1), *CCR2* C-C chemokine receptor type 2, *GA* glatiramer acetate, *GFP* green fluorescent protein, *M-CSF* macrophage colony-stimulating factor, *MΦ* macrophages, *MG* microglia, *Mo* monocytes, *MOG45D-DC* dendritic cells loaded with altered myelin oligodendrocyte glycoprotein-derived peptide (MOG45D; a weak agonist and a non-encephalitogenic variant of MOG_(35–55)_ peptide), *NEP* neprilysin, *SCARA1* class A1 scavenger receptor, *TGF-β* transforming growth factor-β, *WT* wild type

^a^Increased Mo infiltration per Aβ plaques

^b^ *APOE*-dependent effect

### Microglia-mediated phagocytosis

Microglia aid in the normal development, function, and repair of the CNS. In response to injury or other pathological conditions, microglial processes and cell bodies migrate to lesion sites and initiate an immune response to contain and resolve particular insults [123, 124, 299]. Activated microglia are closely associated with senile plaques in both human and ADtg models. While microglia are capable of clearing Aβ *in vitro* [241, 300, 332–334], their *in vivo* clearance capacity has been questioned [335–337]. Successful Aβ internalization by microglia has been documented in some cases [338, 339], while others report incomplete processing [239, 240, 335–337]. In support of the latter, depletion of microglia in three different ADtg mouse models had no effect on fibrillar or soluble Aβ accumulation, indicating that microglia are not chiefly responsible for Aβ clearance in these models [335, 336]. Moreover, aging and toxic conditions in the AD brain render microglia chronically activated. This further reduces their phagocytic capacity and causes a prolonged neuroinflammatory response, including production of reactive oxygen species (ROS), cytokines [e.g. IL-1β, IL-6, TNFα, and transforming growth factor β (TGF-β)] and chemokines [e.g. macrophage inflammatory proteins (MIPs), monocyte chemotactic protein 1 (MCP-1), and C-C chemokine receptor types 3 and 5 (CCL3 and CCL5)] [340–342]. Elevated levels of these mediators have potent neurotoxic effects [343, 344] and correlate with increased Aβ pathology in certain brain regions of human AD patients and transgenic murine (APP/PS1) models [345]. Additionally, recent reports showed that microglia continue to participate in synaptic remodeling in aged mice [346], and can exacerbate synaptic dysfunction by modifying dendritic spine density and inappropriately engulfing endangered neurons [310, 347]. The aberrant microglial-mediated engulfment of dysfunctional synapses in ADtg models was mediated by components of the complement cascade (i.e. C1q, C3, CR3). Considering the recent genetic data linking certain SNPs in CR1 to the development of AD (Table 1), this work provides further support for the role of the immune system in AD.

Although this indicates a detrimental role for microglia in the AD brain, plaque-associated microglia have been shown to degrade scar tissue proteins with secreted proteases, clear cellular debris, and recruit the adaptive arm of the immune system to stimulate or regulate effective local immune responses [148, 348]. A recent investigation using *in vivo* two photon imaging also demonstrated that early on, microglia form a protective barrier around developing plaques, preventing accumulation of Aβ_42_ protofibrils and associated local neuritic damage [349]. Remarkably, a recent study demonstrated that stimulating hippocampal interneurons at frequencies consistent with gamma oscillations alters microglial phenotype and behavior in the 5XFAD model [350]. A 1-hour delivery of 40 Hz stimulation lowered global Aβ levels and modified microglial gene expression so that they more efficiently engulfed Aβ. Based on the available evidence, microglia cannot be labeled as either neuroprotective or neurotoxic. Instead, microglia co-exist in a range of functional states: ramified-resting under physiological conditions, classically and alternatively activated in response to injury, or dystrophic and neurotoxic in aging and chronic inflammation. These phenotypes are highly sensitive to the changes in CNS composition that accompany senescence and the neurodegeneration seen in AD [351]. Current research posits that in early stages of disease, healthy microglia comprise the first line of defense in restricting Aβ pathology, effectively clearing fibrillar and soluble Aβ through phagocytosis and proteolytic processing [123]. However, aged and diseased microglia in the AD brain have a markedly reduced capacity to do so [335, 337, 339, 349]. Taken together, it is not surprising that microglia have become candidates for potential disease-modifying therapies.

### Monocyte/macrophage-mediated Aβ phagocytosis

Like microglia, monocyte-derived macrophages are professional phagocytes that support normal tissue function. However, microglial senescence in AD suggests that monocytes may have unique, complementary functions in the disease state, although this conclusion is highly controversial [303, 352–355]. Supporting evidence from genetic and physiological studies of human peripheral blood monocytes (PBMCs) highlights the importance of healthy, functional monocytes in mitigating disease (see summary of studies in Table 3). PBMCs isolated from AD patients exhibit poor differentiation, impaired phagocytosis, and increased pro-inflammatory cytokine production in response to soluble Aβ [125, 127, 229, 243, 315, 356] (Table 3). Further, rare variants of *CD33* and *TREM2*, two genes negatively impacting the phagocytic and Aβ clearance capacity of monocytes, confer a greater risk of developing AD (Table 1) [33, 34, 64, 96, 155]. It remains to be elucidated whether the altered monocyte phenotype is a cause or consequence of disease.

## Receptor-mediated Aβ phagocytosis: molecular machinery

Despite key differences highlighted previously, microglia and monocyte-derived macrophages do overlap in terms of phagocytic receptor expression and behavior [296, 301]. An extensive body of work describes under which conditions and by which mechanisms these cells are capable of engulfing distinct Aβ species. For example, microglia have been shown to phagocytose fibrillar Aβ_40_ and Aβ_42_ under *in vitro* [239, 299, 300, 334, 357], *in vivo* [358, 359], and *ex vivo* experimental conditions [357, 360]. However, the mechanism underlying soluble Aβ uptake is less clear. Some argue that microglia phagocytose soluble Aβ_42_ as they do fibrillar forms [332, 361], while others suggest uptake occurs through fluid-phase macropinocytosis [333]. These two processes may not be mutually exclusive; more precise methods of isolating distinct soluble oligomeric forms may reveal assembly dependent interactions with microglia. Similarly, studies using PBMCs isolated from healthy patients have demonstrated the ability of monocytes to effectively bind [356] and engulf soluble and fibrillar Aβ_42_ [125, 185, 315]. The following sections describe the major phagocytic receptors engaged in myeloid cell-mediated physiologic clearance of Aβ. Whenever possible, the discussion delineates between uptake of soluble oligomeric Aβ_42_, fibrillar Aβ_42_, and other conformations or alloforms. This distinction is particularly relevant given the varying toxicities of different Aβ species.

### Toll-like receptors (TLRs)

TLRs are a family of pattern recognition receptors with distinct functions in the innate immune response. TLR2 and TLR4, in particular, were shown to be indirectly involved in Aβ phagocytosis through the formation of a receptor complex with CD14 and the subsequent activation of microglia and monocytes. Inhibiting or deleting any component of the CD14-TLR receptor complex in human monocytes or murine microglia diminished the production of pro-inflammatory cytokines and phagocytosis of fibrillar Aβ_42_ [362, 363].

### Macrophage scavenger receptor 1 (SCARA1)

SCARA1 (alternatively named MSR-1, CD204, type-A1 scavenger receptor, and SR-A) is one of the principal receptors involved in Aβ uptake by immune cells. It is expressed on human and rodent macrophages [364], microglia [299, 332], and human monocytes [365]. SCARA1 can bind both soluble and fibrillar Aβ_42_ *in vitro* [326, 332, 365] and facilitate its subsequent uptake. Lack of functional SCARA1 in murine microglia and monocytes reduced Aβ_42_ uptake by a range of 50%-65% in several experimental preparations [326, 366]. Glatiramer acetate (GA), an altered myelin-derived antigen with demonstrated immunomodulatory benefits in ADtg mice [9, 144, 148, 348, 367], was shown to upregulate surface expression of SCARA1 on monocyte-derived macrophages and to increase Aβ uptake by this cell population [144]. Immunization with the FDA approved drug, GA, is an intriguing therapeutic strategy and will be discussed further below.

The importance of SCARA1 function in Aβ clearance has also been established *in vivo*. SCARA1-deficient APP_SWE_/PS1_ΔE9_ transgenic mice exhibited increased mortality and a significant elevation in surface area fraction stained for Aβ compared to control ADtg mice [326]. Increased microglial expression of SCARA1 around Aβ plaques has been demonstrated in multiple ADtg models [368, 369] as well as in human AD brains [370]. SCARA1 expression on CNS phagocytes appears to have a neuroprotective role in restricting toxic forms of Aβ and mitigating disease progression.

### CD36

CD36 is a type B scavenger receptor expressed on the cell surface of monocytes, macrophages, astrocytes, and neurons [371]. CD36 has been shown to mediate phagocytosis of fibrillar Aβ_42_ through interactions with two distinct receptor complexes acting as a functional unit [334, 368, 371]. CD36-deficiency prevents microglial accumulation in response to stereotaxic intracerebral injections of fibrillar Aβ [372], and antagonists of CD36 effectively block phagocytosis of fibrillar Aβ_42_ in microglia cell lines [334]. Like SCARA1, expression of CD36 is substantially increased in monocyte-derived macrophages in response to GA stimulation, which may contribute to their superior Aβ clearance ability compared to untreated macrophages [144] (Fig. 1b; Table 4). CD36 was also shown to bind soluble Aβ_42_ directly [361, 373], although it may play a redundant role in soluble Aβ_42_ clearance [326]. Specific knockdown or inhibition of CD36 demonstrated a sustained ability of microglia to phagocytose soluble Aβ_42_ with continued expression of other scavenger receptors [332, 361].

CD36 adequately demonstrates the dichotomous role of microglia in AD pathogenesis. While CD36 confers neuroprotection through induction of Aβ removal, it also activates the NLRP3 inflammasome in microglia and stimulates pro-inflammatory cytokine release (i.e. interleukin IL-1β and ROS). Thus, microglia may contribute to the toxic environment that induces their own impairment [334, 361, 373, 374]. Moreover, a recent study has demonstrated that the soluble Aβ_42_-induced inflammatory milieu directly inhibits microglial phagocytosis of Aβ_42_ fibrils and downregulates CD36 expression *in vitro* [374]. In sum, it seems the ability of CD36 to initiate Aβ uptake is differentially regulated by multiple toxic species that accumulate in AD brains.

### TREM2

The triggering receptor expressed on myeloid cells 2 protein is a single-pass type 1 transmembrane protein that is part of the immunoglobulin superfamily. Ligands of this receptor include anionic carbohydrates, phospholipids, and apolipoproteins such as ApoE [375–377]. TREM2, along with the protein DAP12, forms a signaling complex that is responsible for the activation of immune responses in myeloid cells including microglia, macrophages, and monocytes [378]. In AD, however, the predominant TREM2-expressing cell type has been contested [324, 376].

GWASs have recently implicated the R47H variant of *TREM2* as an AD risk factor in multiple populations [96, 155]. In a post-mortem analysis of AD and control brains with and without the R47H variant, the mutation was associated with greater levels of pro-inflammatory markers and increased amyloid load in all brain areas examined [102]. Other *TREM2* risk alleles have also been identified, including R62H and D87N [96, 155]. Remarkably, these mutations and others occur exclusively in the ligand-binding domain of the protein and diminish affinity of the mutant TREM2 to its ligands [377]. It was further demonstrated that myeloid cells can clear Aβ directly through TREM2-mediated uptake of lipoprotein-Aβ complexes, modeling the ApoE-Aβ interactions observed *in vivo* [377]. Moreover, monocytes isolated from AD patients with the R62H variant were unable to clear lipoprotein-Aβ complexes as efficiently as healthy controls. These findings imply that microglia and monocytes require a functional TREM2 protein to appropriately phagocytose Aβ.

Studies utilizing ADtg mouse models, however, point to a much more complex role for TREM2 than previously thought [323, 324, 376]. In one study, *TREM2* knockout in APP/PS1 mice greatly ameliorated disease progression [324], while two other investigations successfully demonstrated TREM2-expressing immune cells containing AD pathology [323, 376]. The evidence appears contradictory; however, TREM2-modulated neuroinflammation and Aβ clearance may be highly context-dependent, influenced by the immune cell type and the inflammatory milieu in which it is expressed. In light of this, a recent study has shown that TREM2-deficient microglia and monocyte-derived macrophages phagocytose less fibrillar Aβ_42_ compared to wildtype cells, an impairment partially rescued by therapeutic anti-Aβ antibodies. Antibody-coated Aβ greatly enhanced phagocytosis by both *TREM2* knockouts and wildtype cells, although clearance by mutant cells lagged behind controls under all conditions [325]. This finding has important implications for the efficacy of Aβ-targeted immunotherapies in patients with *TREM2* mutations, yet further research is needed to fully elucidate these relationships.

### CD33

CD33 is a member of the sialic acid-binding immunoglobulin-like lectins (SIGLECS) family, expressed on myeloid cells [65, 379]. In general, it is thought to dampen the immune response perhaps by inhibitory signaling through immunoreceptor tyrosine-based inhibition motifs (ITIM) [380]. In the brains of AD patients, CD33-positive microglia are enriched relative to age-matched controls and correlate with greater Aβ_42_ levels and plaque burden [65]. The diminished capacity of CD33-expressing microglia to phagocytose Aβ_42_ is thought to explain this relationship. In support of this, possession of the newly discovered rs3865444^C^ risk allele [33, 34] results in a sevenfold increase in CD33 expression on monocytes with a significant reduction in ability to phagocytose Aβ_42_. Monocytes isolated from young individuals with the rs3865444^C^ risk allele also displayed an Aβ_42_ phagocytic deficit [64]. Enriched monocytic CD33 expression actually mediated the relationship between this risk allele and higher amyloid plaque burden in AD brains [64]. Conversely, the protective rs3865444^A^ allele dampens CD33 expression and increases the proportion of CD33 molecules that lack a SIGLEC-specific region responsible for phagocytosis inhibition [379]. These findings provide proof of impaired monocyte-mediated interactions with Aβ and enhanced disease risk. AD-related immune deficits are thus not solely driven by senescence or the disease process itself. Rather, monocyte phagocytic impairment may far precede Aβ deposition, as seen in these cases, and arguably predisposes to greater amyloid accumulation and lifetime risk.

## Role of monocytes in AD: evidence and controversy

Despite the surging data favoring a critical role of monocytes in AD pathophysiology, it is important to acknowledge the contradictory evidence in the field surrounding monocyte-mediated Aβ clearance in chronic neurodegenerative diseases. Major questions remain. (1) Under what conditions do monocytes infiltrate the CNS? (2) Do monocytes and macrophages behave differently from microglia once in the CNS parenchyma, especially in their ability to resist misfolded Aβ forms? (3) Is the neuroprotection exhibited by monocytes a predominantly peripheral blood or a local effect? And (4) Is the effect cell-mediated, molecular or plasma-mediated, or both? The following sections address these controversies given the available literature and identify methodological discrepancies that may have generated some confusion.

### Cerebral infiltration of monocytes in murine models of Alzheimer’s disease

Monocyte infiltration in AD was first documented by seminal studies transplanting GFP-labeled bone marrow cells into irradiated ADtg mice [295–297, 318]. Monocytes were shown to preferentially home to Aβ plaques and participate in their clearance [295–297, 318]. The applicability of these studies to normal physiology was later questioned due to the use of whole body irradiation (including brain) and bone marrow transplantation; the former in particular may artificially enhance monocyte infiltration into the brain parenchyma [355, 381]. Specifically, irradiation is known to induce transient BBB leakage, permitting greater passage of cells and blood contents. In addition, whole marrow transplantation increases the number of progenitor cells in the circulation.

To further elucidate the effects of irradiation, the GFP-transplantation paradigm was repeated, this time shielding the heads of recipient mice to conserve BBB integrity. This procedure reduced monocyte infiltration into the CNS, and called into question the conditions necessary for monocyte recruitment [330]. However, several investigations have successfully demonstrated spontaneous monocyte infiltration in the absence of irradiation, genetic manipulation, or chemotherapy (Table 4) [144, 146]. These experiments enriched the peripheral circulation with either CD11b^+^ or CD115^+^ monocytes from the bone marrow of young adult wildtype mice, rather than whole blood marrow, eliminating the additional confounder of increased progenitor cell numbers seen in earlier studies. Importantly, blood enrichment with GFP monocytes in age-matched wildtype (non-ADtg) animals did not cause recruitment of monocytes to the CNS [144, 146], implicating that a diseased-brain is a precondition for their cerebral recruitment. Taken together, brain irradiation is neither necessary nor sufficient for monocyte recruitment. Rather, several other conditions are consistently required, at least in ADtg models—namely, the presence of amyloid pathology, especially soluble oligomeric or fibrillar Aβ_42_ forms [382, 383], and binding of the monocytic surface receptor CCR2 to its ligand, MCP-1 [126, 298, 384, 385].

The mechanism by which cerebral amyloid accumulation induces monocyte infiltration is multifactorial. Vascular Aβ deposition can directly damage the vessel wall [386] and allow greater passage of monocytes into the parenchyma. Indeed, the presence of a leaky BBB was confirmed in 40–60% of AD patients [387, 388]. Furthermore, the Aβ-induced immune response alters the expression and production of inflammatory cytokines, chemokines, and their receptors [123, 311, 340–342]. The expression of MCP-1, a critical signaling factor for monocyte recruitment, is upregulated near Aβ plaques, on microglia, and on microvessels in the brains of AD patients and ADtg mice [127, 384, 385]. It is therefore postulated that the AD brain, and specifically chronically activated and overwhelmed microglia, solicit additional assistance from peripheral monocytes through MCP-1 signaling [126, 144, 148, 191, 297, 311, 389]. Other signaling cascades remain poorly understood.

### Depletion or enrichment of myeloid cells: impact on cerebral Aβ burden

Modulation of monocyte recruitment to the CNS clearly demonstrates the significant contribution of monocyte-derived macrophages to Aβ clearance. Blocking CCR2 signaling [298, 330, 331] or selectively ablating these cells in the blood [296, 297, 328] greatly accelerates Aβ accumulation in ADtg models. Conversely, inducing monocyte recruitment by lipopolysaccharide (LPS) stimulation, immunization, or monocyte engraftment significantly reduces parenchymal and vascular amyloid pathology in transgenic mice [144–146, 148]. These investigations coupled with compelling *in vitro* data [144, 241] led to the conclusion that monocyte-derived macrophages, compared to their resident counterparts, possess a superior ability to clear fibrillar Aβ in AD (Fig. 1), resolving inflammation in spite of the toxic environment [240, 241, 296, 297, 300, 390].

Other studies utilizing microglial ablation techniques challenge this assumption. Crossing ADtg mice with the *CD11b-HSVTK* model, in which the thymidine kinase of the herpes simplex virus is expressed under the *CD11b* promoter, allows for elimination of local, proliferating myeloid cells upon intracerebroventricular administration of ganciclovir. Peripheral GFP-labeled macrophages can then repopulate the CNS, introduced by either transplantation [354] or parabiosis with an actin-enhanced GFP partner [353]. In both cases, repopulation did not augment plaque burden, insoluble Aβ, or soluble forms. Importantly, macrophages were diffusely spread across the parenchyma, in stark contrast to the plaque-associated microglia of control mice and the demonstrated plaque-homing abilities of monocytes in other models [353, 354]. Given the inability of re-populating monocytes to clear Aβ, these studies concluded that monocytes do not play a significant role in restricting amyloid pathology. However, it is possible that microglial depletion critically alters the delicate milieu required to induce monocyte phagocytic and anti-inflammatory properties. Indeed, the interplay between microglia, astrocytes, monocytes, and molecular mediators such as scar tissue proteins [i.e. chondroitin sulfate proteoglycans (CSPGs)], has been shown to attract these cells to the lesion sites and induce phenotypic shifts needed for protection in various disease states [144, 145, 148, 191, 297, 348, 367, 391]. Specifically, senescent, plaque-associated microglia are known to release MCP-1 required for monocyte recruitment [126, 298, 384, 385, 389, 392]. In addition, the impact of ganciclovir-induced neurotoxicity is poorly understood. From these repopulation studies, it is apparent that elimination of microglia impacts monocyte phenotype and function, and as such, these findings may not be representative of monocyte behavior in the natural progression of disease.

It is undeniable though that certain conditions do in fact enhance the migratory and Aβ clearing capacity of infiltrating monocytes over their resident counterparts. In particular, ADtg mice immunized with the myelin-derived peptides MOG45D or GA exhibited reduced Aβ levels and neuroinflammation, attributable to the increased recruitment of anti-inflammatory monocytes that directly engulfed Aβ [144, 148]. Other immunomodulatory approaches involving targeted overexpression of Aβ-degrading enzymes to [145, 146, 191] or genetic manipulation of [322] peripheral monocytes have demonstrated similar monocyte-mediated abrogation of Aβ deposition [Table 4]. These interventions may form the basis of promising, disease-modifying therapies that will be discussed further below.

### Peripheral effects of monocytes on Aβ clearance

Recognition of the heterogeneity of different monocyte subtypes has emerged from recent studies that identified new functional biomarkers for myelomonocytic cells. An immunohistochemical and activity-based distinction has been proposed between murine monocyte subsets: an inflammatory (Ly6C^hi^CX3CR1^int^CCR2^+^) type pertaining to CNS recruitment and parenchymal Aβ clearance, and a patrolling (Ly6C^lo^Cx3CR1^high^CCR2^−^) type that remains associated with the vasculature [303, 308]. The discussion thus far has exclusively focused on the local effects of the inflammatory subset and their ability to reduce cerebral Aβ load in the parenchyma through cellular uptake and enzymatic degradation. However, mounting evidence suggests an additional role for patrolling monocytes and perivascular macrophages in the regulation of cerebral amyloid angiopathy (CAA), a disease process in which amyloid plaques accumulate within the walls of small cerebral blood vessels [129, 160]. CAA is seen in over 80% of AD patients and is frequently associated with microhemorrhages and cognitive deficits. Real-time *in vivo* imaging of APP/PS1 mice has elegantly demonstrated that patrolling monocytes are in fact attracted to and crawl along Aβ-positive veins, where they engulf Aβ and subsequently recirculate into the bloodstream [329]. To further confirm their role in perivascular Aβ clearance, depletion of patrolling monocytes substantially increased Aβ levels in the vasculature [328] as well as in the cortex and hippocampus [329]. A proposed equilibrium of Aβ clearance exists between the different CNS-associated compartments, including the brain parenchyma, perivascular spaces, CSF, and peripheral blood [328, 393, 394]. Thus, the recirculation of Aβ-containing monocytes to the periphery may effectively pull other Aβ species out of the parenchyma—a process termed the peripheral sink effect.

In addition to monocytes and macrophages in the perivascular space, recent data suggest that the activity of these cells in the peripheral blood may be pivotal for the regulation of neuroinflammation associated with AD and for inducing neuronal regeneration [144, 148, 163, 348, 367, 395–397]. Murine parabiosis studies, in which the vasculatures of two mice are joined, have effectively illustrated the impact of peripheral immune cells and, moreover, blood-soluble immune mediators on brain health. Joining the vasculatures of wildtype and ADtg mice, either before or after the onset of Aβ deposition, reduced Aβ plaque burden in the cortex and hippocampus of the ADtg parabiont, while also attenuating neuroinflammation, hyperphosphorylated tau, and neuronal apoptosis [397]. This was achieved in the absence of monocyte infiltration or CNS manipulation of known Aβ clearance pathways. It is therefore inferred that effective Aβ removal in murine models can be achieved by several mechanisms: either by blood enrichment of wildtype peripheral monocytes to boost infiltration and clearance of Aβ from brain parenchyma or by replacement and repair of the blood-soluble milieu to induce beneficial phenotypic changes in brain parenchymal cells that promote Aβ clearance. In support of the latter, earlier studies of parabiosis between young and old wildtype mice demonstrated increased synaptic plasticity, neurogenesis, and cognitive capacity in the older parabionts when sharing blood with young mice [395, 396]. This effect was attributed to the specific milieu in the blood of the younger mice rather than infiltration of peripheral immune cells [395]. Indeed, monocytes release small, soluble mediators, such as cytokines and chemokines, which can traverse the BBB and enter the brain parenchyma. Monocytes were also shown to promote anti-inflammatory behavior of surrounding microglia and astrocytes in several other disease models [144, 145, 148, 191, 297, 348, 367, 391]. Further investigation is greatly needed to understand the signaling that takes place in these models.

## Therapeutic effects of peripheral monocytes and macrophages

Given the believed function of monocytes in AD etiology and the ease of access to the peripheral blood, modulation of monocyte phenotype and behavior represents a promising therapeutic target. Though not yet translated into clinical practice, recent investigations in murine models highlight the potential benefit of enhancing monocyte recruitment to the AD brain.

Stimulation with two distinct exogenous compounds has been successful in promoting Aβ clearance. Dietary curcumin, a major component of the spice turmeric, directly interacts with oligomeric and fibrillar Aβ_42_ [398] and may enhance Aβ phagocytosis by human PBMCs [315, 399]. Additionally, injections of the macrophage colony-stimulating factor (M-CSF) into APP_SWE_/PS1 mice prior to signs of cognitive impairment had a number of positive effects. These included increased circulating levels of CD45^+^/CD11b^+^/CD115^+^ monocytes and phagocytic activity of Aβ by Iba-1^+^ immune cells in brain parenchyma [320], leading to decreased size and density of Aβ plaques, and prevention of learning and memory deficits [321].

As mentioned above, peripheral immunization with DCs loaded with MOG45D (MOG45D-DC) or with GA also had profound effects on the function of innate immune cells, which consequently reduced various pathological features of AD. In ADtg mice, MOG45D-DC immunization increased CNS recruitment of anti-inflammatory macrophages, demonstrated by reduced TNF-α and increased IL-10 and TGF-β expression, that efficiently phagocytosed Aβ [148]. As a result, these mice showed restricted vascular and parenchymal Aβ deposits and reduced soluble Aβ_42_ levels, as well as increased expression of the Aβ-degrading enzyme MMP-9. GA immunization of ADtg mice yielded the same beneficial immunomodulatory and plaque-clearing effects [144, 297, 348], while also promoting neurogenesis and preservation of synapses and cognitive function [144]. In agreement with these findings, several other studies have shown that suppression of regulatory T-cells, either via peripheral blockade of the programmed cell death protein-1 (PD-1) [400] or of TGF-β signaling in monocyte-derived macrophages [322], enhanced monocyte recruitment to the brain in ADtg mice, and resulted in Aβ removal and improved cognitive performance [322, 400].

Other methods of immune modulation include adoptive transfer of healthy monocytes and bone marrow transplantation. Infusion of wildtype CD115^+^ monocytes to the peripheral blood of ADtg mice stimulated their spontaneous migration to amyloid lesions in the absence of irradiation, genetic manipulation, or chemotherapy. Treated mice exhibited reduced cerebral Aβ protein levels and astrogliosis, preserved pre-synaptic integrity, and ameliorated cognitive deficits [144]. Likewise, bone marrow transplants from wildtype donors increased monocyte recruitment to the CNS at sites of amyloid accumulation, while also reducing plaque burden [295, 297, 318].

Because peripheral monocytes were shown to cross the BBB and home to sites of Aβ accumulation, they can function as a delivery system of therapeutic agents. Targeted overexpression of either NEP or ACE has proven beneficial in abrogating AD progression in murine models. Injecting 9-month-old ADtg mice with NEP-expressing monocytes completely prevented further Aβ deposition when compared to untreated ADtg mice or those infused with monocytes containing inactive NEP [146]. Similarly, targeted overexpression of ACE to monocytic cells in the bigenic APP/PS1 mouse model of AD markedly reduced both soluble and insoluble levels of Aβ_42_, limited plaques and astrogliosis, and preserved cognitive function [145, 191].

## Conclusion

Aβ clearance is a complex, multifactorial process, requiring the collaboration of various systems and cell types. Aβ can be removed to the peripheral circulatory or lymphatic systems by transport across the BBB or by absorption from the CSF and ISF. While innate immune cells are known to phagocytose and degrade fibrillar Aβ, these cells were only recently shown to engulf and clear soluble Aβ species as well. It is still unclear whether Aβ accumulation is a cause or consequence of disease. However, mounting evidence has shown that increased cerebral Aβ burden is the earliest pathognomonic event in AD. Moreover, soluble, oligomeric Aβ was shown to directly incite nerve and synaptic damage, leading to impaired neuronal function. In the late-onset, common cases of AD, Aβ buildup is attributed to defective clearance, rather than to its overproduction. The observed deficiency could result from impairments in any one of the removal processes or, more likely, a combination of minor clearance deficits and compounding risk factors that varies from patient to patient. Modulation of clearance mechanisms may be an important early strategy for curtailing Aβ accumulation and disease progression.

As our knowledge of AD continues to expand, so does a body of evidence that supports a key role for innate immune cells, especially monocyte-derived macrophages, in Aβ removal, local immune regulation, and repair. Bone marrow-derived monocytes can cross the BBB and clear Aβ through cellular uptake and enzymatic degradation, perhaps even more efficiently than resident microglia. The clearance process is, again, complex. Phagocytosis requires the coordination of many surface receptors (e.g. TLRs, integrins, scavenger receptors) for recognition and uptake, followed by intracellular trafficking, ultimately to lysosomes, for degradation. Monocytes, macrophages, and microglia also mediate extracellular Aβ degradation through surface expression or release of various proteases, such as ACE, IDE, NEP, and MMP-9. These functions were reported to be markedly impaired in peripheral monocytes isolated from AD patients. It is possible that the observed deficiency is a consequence of immune senescence and AD-related degeneration, or perhaps their dysfunction is a direct contributor to disease development. In support of the latter, possession of a rare variant of the AD-associated *CD33* gene impacts the phagocytic capacity of monocytes isolated from young adult patients, indicating that this particular functional deficit is present throughout life. Other GWAS data have linked multiple immune-related risk factors to AD. Known relationships between the major risk gene *TREM2* and monocyte/microglia phagocytic function offer a compelling demonstration of the immune system’s impact in AD.

Aggregates of misfolded Aβ are known to trigger a prolonged neuroinflammatory response that is tightly associated with synaptic dysfunction and cognitive decline. Enhancing cerebral recruitment of monocytes through either peripheral infusion or immunization with altered myelin-derived antigens was shown to temper these degenerative changes in murine models. Specifically, monocytes were able to efficiently clear Aβ and resolve the resulting astrogliosis and neuroinflammation, thereby preserving synaptic integrity and cognitive function. Immunomodulation approachs that enhance cerebral recruitment of neuroprotective monocytes hold great promise as disease-modifying therapeutic interventions and represent a valuable target for further application and translation.

## Abbreviations

AD: Alzheimer’s disease
ADtg: AD transgenic models
Aβ: Amyloid-β protein
AICD: Amyloid intracellular domain
APP: Amyloid precursor protein
ACE: Angiotensin-converting enzyme
ApoE: Apolipoprotein E
ApoER2: ApoE receptor 2 A
AQP4: Aquaporin 4
ABC: ATP-binding cassette
AV: Autophagic vacuole
BACE1: β-secretase 1
BBB: Blood–brain barrier
CCR2: c-c chemokine receptor type 2
CNS: Central nervous system
CAA: Cerebral amyloid angiopathy
CSF: Cerebrospinal fluid
CMA: Chaperone-mediated autophagy
ECE-1: Endothelin-converting enzyme 1
FAD: Familial Alzheimer’s disease
GWAS: Genome-wide association study
GA: Glatiramer acetate
GFP: Green fluorescent protein
HLA: Human leukocyte antigen
pTau: Hyperphosphorylated tau
ITIM: Immunoreceptor tyrosine-based inhibition motif
IDE: Insulin-degrading enzyme
IL-1β: Interleukin-1β
IL-10: Interleukin-10
ISF: Interstitial fluid
Iba1: Ionized calcium binding adapter molecule 1
LOAD: Late-onset AD
LPS: Lipopolysaccharide
LRP-1: Low-density lipoprotein-related protein 1
LDLR: Low-density lipoprotein receptor
M-CSF: Macrophage colony-stimulating factor
SCARA1: Macrophage scavenger receptor 1
mTOR: Mammalian target of rapamycin
MMP-9: Matrix metalloproteinase-9
MCP-1: Monocyte chemotactic protein 1
MOG45D: Myelin oligodendrocyte glycoprotein-derived peptide
NEP: Neprilysin
NFTs: Neurofibrillary tangles
PBMC: Peripheral blood monocyte
PICALM: Phosphatidylinositol binding clathrin assembly protein
PS1;PS2: Presenilin-1 and -2
ptau: Hyperphosphorylated tau protein
ROS: Reactive oxygen species
RAGE: Receptor for advanced glycation end products
RFP: Red fluorescent protein
CD33: Sialic acid-binding immunoglobulin-like lectin 3
SNP: Single nucleotide polymorphism
TLR: Toll-like receptor
TGF-β: Transforming growth factor β
TREM2: Triggering receptor on myeloid cells 2
TNFα: Tumor necrosis factor
UPS: Ubiquitin–proteasome system
VLDLR: Very low-density lipoprotein receptor
